# Assessing the knowledge and practices of smallholder pig farmers and associated risk factors for swine gastrointestinal disorders in Masindi district, Uganda

**DOI:** 10.1186/s12917-025-04667-2

**Published:** 2025-03-31

**Authors:** Samuel Majalija, Gabriel Tumwine, Juliet Kiguli, Benard Owori, Robert Alex Isabirye, Peter Waiswa

**Affiliations:** 1 Department of Biosecurity, Ecosystem Health and Veterinary Public Health, College of Veterinary Medicine Animal Resources and Biosecurity, Makerere University, P.O Box 7062, Kampala, Uganda; 2 School of Public Health, College of Health Sciences, Makerere University, P.O Box 7062, Kampala, Uganda; 3 Mukono Zonal Agricultural Research and Development Institute of the National Agricultural Research Organization (NARO), Kampala, Uganda

**Keywords:** Biosecurity, Diseases, Husbandry practices, Gut, Piggery smallholder farmers

## Abstract

**Background:**

Piggery production is a main income source for the rural poor in Uganda, where 1.3 million households own about 4.47 million pigs. Nonetheless, health challenges and lack of knowledge by the farmers affect the productivity and profits of the pig enterprises. Thus, this study determined the knowledge and practices among smallholder pig farmers and the associated risk factors for pig gastrointestinal disorders in Masindi district, Uganda.

**Methods:**

A cross-sectional survey was conducted using a closed-end questionnaire interview of 170 smallholder pig farmers in the rural district of Masindi, from March to July 2020. The qualitative data was analyzed and presented as frequencies, percentages, and their 95% confidence intervals. Bivariate and multi-variate analysis were used to determine factors associated with GIT disorders.

**Results:**

Of the 170 farmers, males and females were equal (50%), mostly as pig owners (91.2%), rural folks (88.8%), who raised other animal species (72.9%) and with 5–10 years (72.4%) of experience. Of these, 67.6% were married, mainly in age ranges of groups 19–50 (90.6%) and 47.6% had attained primary school level. Pig rearing was for income generation (95.5%) and 79.4% reported GIT disorders on their farms, affecting local breeds (57.0%) of all age groups and throughout the year. Similarly, most of the farmers understood transmission routes of GIT disorders and adhered to biosecurity practices. However, 62.9% treated the sick pigs, 40.6% sold the sick pigs and only 25.9% consulted a veterinarian. At multivariable logistic regression analysis, being a female farmer (*p* = 0.018, OR = 3.163, CI: 1.213, 8.244); mixing of different herds of pigs (*p* = 0.003, aOR = 4.141, 95%CI (1.317,13.013); feeding pigs on raw tubers (*p* = 0.017, aOR = 2.703, 95% CI (1.198,6.099) and scavenging (*p* < 0.001, aOR = 9.605, 95% CI (2.131,43.289) were significantly associated with GIT disorders.

**Conclusion:**

Poor husbandry practices especially feeding on raw tubers, scavenging and mixing of different herds of pigs were associated with widespread pig GIT disorders. Involving women in strategies to improve pig GIT health as equal partners is suggested. Farmers are encouraged to adopt confinement and housing of pigs as the minimum good husbandry practices for sustainable pig production.

**Supplementary Information:**

The online version contains supplementary material available at 10.1186/s12917-025-04667-2.

## Introduction

Agriculture, Uganda’s main economic activity employs more than 65% of the population (45 million), with over 1.34 million households engaged in the pig value chain [1]. The current pig population of about 4.47 million ranks piggery as the third priority livestock sector after goats and cattle [2]. Generally, pig production is attractive to smallholder farmers who raise less than 25 pigs on free range, ropes, or under semi-intensive confinement [3]. Pig keeping plays a crucial role in generating incomes and ensuring nutritional security for rural households [4–6]. It is supported by the abundant local feed resources, small capital investment and faster financial returns due to the short reproduction cycle [7]. The government of Uganda has therefore selected piggery as a potential sector to improve incomes for the rural poor households [4]. Moreover, taking advantage of the high pork demand and consumption rate in Uganda, averaging 3.4 kg/person per year, the highest in East Africa and second in African [8] is a big boost to the sector. Nonetheless, the piggery sector is beset by health challenges, poor husbandry practices, feeding costs and generally poor knowledge among the key players in the pig value chain [7].

The gastrointestinal tract (GIT) plays a critical role in maintenance of the health and productivity of the pig [9, 10]. It contributes at least 70% of the immune cells for mounting an immune-response against ingested foreign agents that end up in the lumen, secretes digestive enzymes and absorbs nutrients [10–13]. Normally, the GIT is colonized by commensal microflora including Lactobacillus, which sustain a balanced microbial ecosystem [14, 15, 13] for optimal metabolism, nutrient utilization and rapid growth rates [16–19], hence higher returns for the pig farmers. However, a shift in the Lactobacilli population structure, either through a change in the GIT ecosystem or intrusion by pathogenic organisms alters the homeostatic balance, with subsequent ensuing GIT disorders [9, 10, 13]. Commonly, the disorders manifest as gas bloating, constipation, ulcerations, erosions, diarrhea and loss of appetite [20], associated with chronic illness, unthrifty malnourished pigs, retarded growth and high mortality rates especially among piglets [14, 16, 21–23].

Collaborative engagement between researchers and farmers is needed for effective prevention and control of GIT disorders and other associated health challenges in piggery. Through knowledge, attitudes, and practices (KAP) studies, the knowledge gaps of smallholder farmers and the associated factors would be identified. Moreover, several KAP studies focusing on the selected swine diseases in Sub Saharan African are available; including African Swine Fever in Nigeria [24], porcine cysticercosis in Tanzania [25]; as well as African Swine Fever [26] and *Taenia solium* in Uganda [27]. Nonetheless, data on the KAP studies associated with gastrointestinal disorders or diseases of pigs in Uganda is lacking. Thus, this study aimed to describe the knowledge and practices among smallholder pig farmers and the associated risk factors for pig GIT disorders in Masindi district, Uganda. Understanding the pig GIT disorders from the farmers’ perspective and the risk factors will guide in the design of effective control and preventive measures among smallholder farmers in Uganda.

## Materials and methods

### Study area

The district is located between latitudes 10 22' and 20 20' north of the equator and longitudes 310 22' and 320 23' east of Greenwich, with an average elevation of 1295 m above sea level. The district's headquarters are located 216 km from Kampala, the nation's capital city, and are situated in Midwestern Uganda. More details of the study area are described elsewhere [28]. The primary economic activity in the district is agriculture and about 80% of households, are engaged in both growing of crops and rearing of livestock. Piggery is a major source of income for more than 60% of households in the villages, which is why this rural district was selected. Pigs and pig products are in greater demand as a result of the region's booming new markets, which are supported by the oil industry.

### Study design

A cross-sectional survey was conducted in the rural district of Masindi from March to July 2020. In consultation with the local veterinarian and farmer leaders, in the subcounty of Bwijanga, which is home to over 10,600 pigs, was selected from 6 rural sub-counties of Masindi district, targeting the relatively higher numbers of pigs, and ease of access of the rural farmers (http://npa.go.ug/wp-content/uploads/2017/05/MASINDI-DDP-FY-2015_2016-_-2019_2020-Final-Copy.pdf).

### Sample size and sampling strategy

The sample size was calculated at a 95% confidence level with the assumed prevalence of the GIT disorders at farm level of 51.7% [3] and margin of error at 7.51% which gave a sample size of 170 smallholder farms from Bwijanga subcounty. The pig-keeping households were established with the help of the veterinary extension staff and the local village leaders, as there were no official records of farmers engaged in piggery. A non-probability snowball sampling method was used to select pig keeping households. With the help of the local veterinarian, the first farmer was selected, and this led to the next immediate farmer. A next household was chosen in case there was no one present or willing to be interviewed. Despite being non-probabilistic, snowball sampling method is recommended for the recruitment of hard-to-reach stakeholders or when there is no prior knowledge about the study subjects.

### Household questionnaire

The study used a closed-end questionnaire (supplementary file 1) in a face-to-face interview with a member of the selected household, who was actively involved in the daily management of the pigs. The questionnaire was pretested on 10 individuals selected from pig keeping households in the neighboring sub county of Budongo. The pretested households were not included in the investigation. The questionnaires were administered by a research assistant conversant with the local Runyoro language used in the area, from March to July 2020. At the start of the interview, the respondents were informed of the objectives of the study and an oral consent to participate in the study was obtained from each farmer. The researcher shared the common signs and symptoms associated with swine GIT disorders to guide the farmers in answering the questions.

The questionnaire was designed to assess the farmers’ knowledge, practices and risk factors associated with pig GIT disorders. The Risk factors considered were divided into i) demographics: gender, religion, level of education level, marital status, residence, activities in the value chain and duration of rearing pigs; ii) general farm practices: breed of pigs kept, ownership, management system, production system, mixing between herds of pigs, sharing of a breeding boar, type of housing system and; iii) feeds and feeding practices: feeding on raw tubers, cooked, dried feeds; pigs scavenging for food, supplement with household food left overs, swill feeds from hotel, feeds from the factory or others feeds to be specified.

### Data analysis

The questionnaire data was entered into excel worksheet (Microsoft Excel for windows, 2013). It was then analyzed using the Statistical Package for Social Science (SPSS version 27) to generate descriptive statistics in form of frequencies and percentages which were presented as tables and charts. The percentages and their 95% confidence intervals (95% CI) were calculated to determine the influence of the participants’ knowledge and practices towards pig GIT disorders in Masindi district. Questions to determine the farmers’ knowledge considered the clinical signs and modes of disease transmission. Questions on practices assessed biosecurity measures undertaken by farmers to prevent and control GIT disorders.

Bivariate analysis was carried out using Fisher’s exact test and binary logistic regression to evaluate the association between the occurrence of GIT disorders and the corresponding potential factors. Odds ratios and 95% confidence intervals (CIs) were calculated, and factors with p values ≤ 0.05 were considered statistically significant. For the selection of independent variables for inclusion in the initial multiple logistic regression model, the entry criterion was fixed at *p* value ≤ 0.20. The model was investigated for interactions and confounding. The fit of the model was assessed using Hosmer and Lemeshow goodness-of-fit test. The model was developed by a stepwise forward selection approach, dropping the least significant independent variable until all the remaining predictor variables were significant (*p* < 0.05).

### Ethical consideration

The study was conducted in accordance with the Declaration of Helsinki, and the protocol was approved by the Ethics Committee of Makerere University, College of Veterinary Medicine, Animal Resources and Biosecurity, ethical approval number MakSBLSREC-2020–031. All participants gave their informed verbal consent in the local language, before taking part in the study. The ethics committee approved the verbal consent because the research was associated with low risk and minimal harm to the participants. The consent was obtained after explaining the research to the participants using an information sheet to guide the verbal explanation of the study. All information collected was kept confidentially; no names or other identifying information was asked during data collection. They were informed that their participation was voluntary and their refusal would not result in any negative consequences. Administrative clearance was obtained from Masindi District Local Government.

## Results

### Socio-demographic characteristics of the participants

A total of 170 household farmers were involved in this study, including an equal proportion of males and females (50%; CI: 42.3–57.8), slightly more (43.5%; CI: 36.0–51.3) were protestants and most had primary school level of education (47.6%; CI: 40.0–55.4) (Table 1).

**Table 1:** Demographic characteristics of the participants

Variable	Category	Frequency (*N* = 170)	Percentage%	Confidence Interval (CI) 95%
Sex	Male	85	50	42.3–57.8
	Female	85	50	42.3–57.8
Religion	Catholic	68	40	32.9–47.8
	Protestant	74	43.5	36.0–51.3
	Others	28	16.5	11.2–23.02
Education Level	None^a^	12	7.1	3.70–12.0
	Primary	81	47.6	40.0–55.4
	O' Level	49	28.8	22.2–36.3
	A' Level	11	6.5	03.3–11.3
	Diploma Holder	8	4.7	2.10–9.10
	Degree holder	9	5.3	2.50–9.80
Age group (Years)	1819	14	8.2	4.1–12.4
	19–30	82	48.2	40.0–55.3
	31–50	72	42.4	35.3–50.0
	> 50	2	1.2	0.0–2.9
Experience in piggery farming (Years)	< 5 years	5	2.9	0.6–5.9
	5–10 years	123	72.4	65.3–78.8
	> 10 years	42	24.7	18.8–31.2
Marital Status	Single	35	20.6	14.8–27.5
	Widowed	12	7.1	3.70–12.0
	Married	115	67.6	60.1–74.6
	Divorced	8	4.7	2.1–09.1
Residence	Rural	151	88.8	83.1–93.1
	Urban	19	11.2	6.90–16.9
Occupation in the value chain	Farmer/owner	155	91.2	85.9–95.0
	Laborer	15	8.8	5.02–14.1
Keep other animals	Yes	124	72.9	65.6–79.5
	No	46	27.1	20.5–34.4

^a^Although the participants lacked formal education, they could read and write in their native language

The age groups mainly engaged in pig rearing were the 19–30 years (48.2%; CI: 40.0–55.3) and 31–50 years (42.4%; CI: 35.3–50.0) more than < 19 years (8.2%; 4.1–12.4) and > 50 years (1.2%; CI: 0.0–2.9). A majority had experience of 5–10 years (72.4%; CI: 65.3–78.8), were married (67.6%; CI: 60.1–74.6), rural folks (88.8%; CI: 83.1–93.1), engaged as pig owners (91.2%; CI: 83.1–93.1), and also reared other animals’ species (72.9%; CI: 83.1–93.1) (Table 1).

**Fig. 1:**
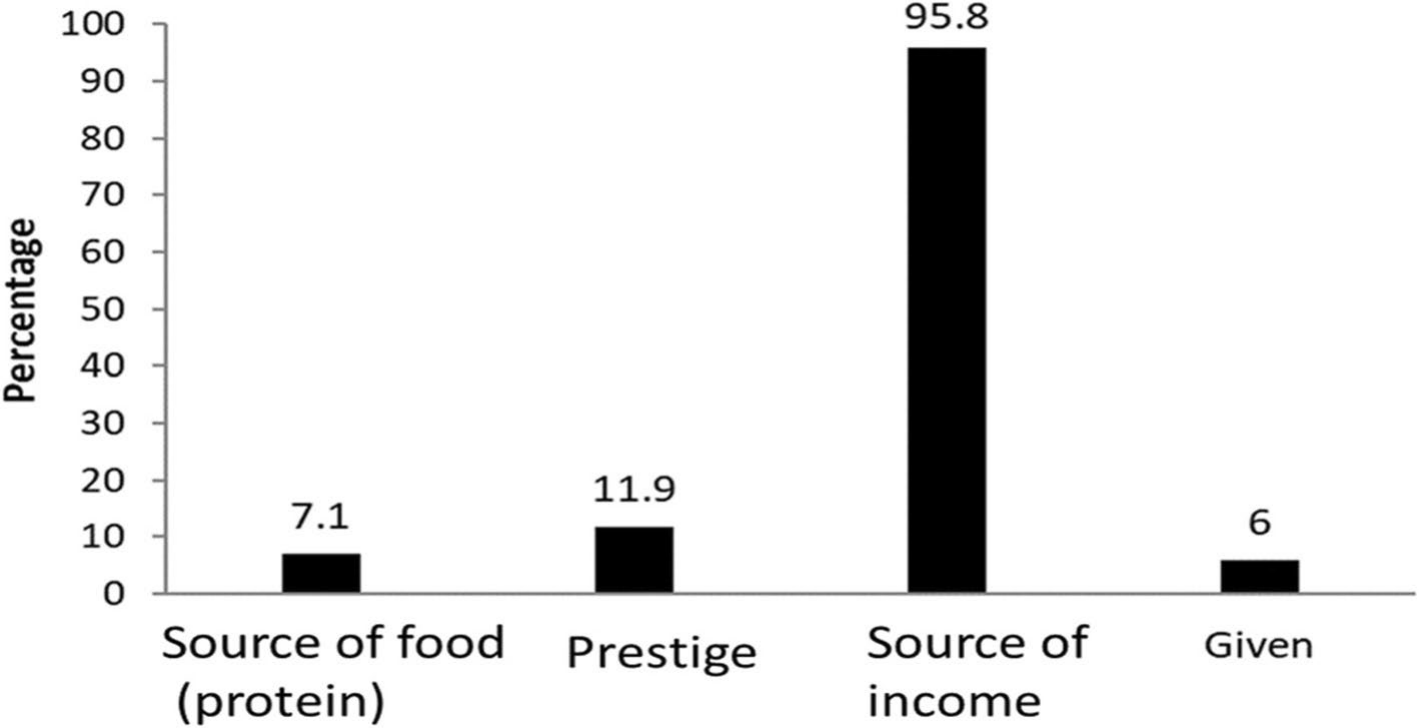
Reasons for rearing pigs by farmers from Masindi

A majority of smallholder farmers reared pig for income generation (95.5%), more than prestige (11.9%) or source of food (protein) (7.1%) as in Fig. 1.

### Determination of knowledge and practices as regards pig GIT diseases among farmers in Masindi district

The knowledge of smallholder farmers was assessed basing on the reported occurrence of GIT disorders, clinical signs and routes of GIT disease transmission. Also, measures for prevention of GIT disorders practiced by the farmers were determined.

#### Prevalence of gastrointestinal tract diseases of pigs and associated husbandry practices

As shown in Table 2, a majority of farmers (79.4%; CI: 72.7–84.8) had experienced gut diseases on their farms in the last 3 months, affecting mainly the weaners (40.7%; CI: 32.8–49.2) and piglets (36.3%; CI: 28.7–44.7) than growers (23%; CI: 16.6–30.8); and disorders occur throughout the year, (38.5%; CI: 30.7–46.9) in the dry season, (30.4%; CI: 23.2–38.6) in the rainy season) and 31.1% (CI: 23.9–39.4) in both seasons. Significantly, a higher percentage of local breeds (57.0%; 48.6–65.1) were reported with GIT disorders than the exotic (14.8%; 9.72–21.9) and mixed breeds (28.1%; 21.2–36.3).

**Table 2:** Prevalence of gastrointestinal tract disorders of pigs and husbandry practices

Attribute	Response	Frequency	Percent (%)	Confidence Interval (CI) 95%
Experienced Gut disease on farm (*n* = 170)	Yes	135	79.4	72.7–84.8
	No	35	20.6	15.2–27.3
Age group commonly affected (*n* = 135)	Piglets	49	36.3	28.7–44.7
	Weaners	55	40.7	32.8–49.2
	Growers	31	23.0	16.6–30.8
Breed commonly affected (*n* = 135)	Local	77	57.0	48.6–65.1
	Exotic	20	14.8	9.72–21.9
	Mixed	38	28.1	21.2–36.3
Common season for occurrence of GIT diseases (*n* = 135)	Dry	52	38.5	30.7–46.9
	Rainy	41	30.4	23.2–38.6
	Both	42	31.1	23.9–39.4

#### Clinical signs associated with GIT disorders as reported by the farmers

**Fig. 2:**
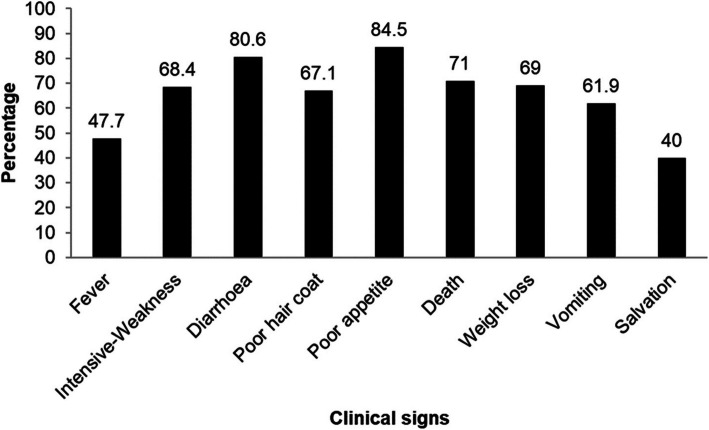
Clinical signs associated with swine diseases identified by farmers

Most farmers were able to identify common clinical signs associated with GIT disorders; poor appetite (84.5%), diarrhea (80.6%), vomiting (61.9), weakness (68.4), weight loss (69%), death (71%) as in Fig. 2.

**Fig. 3:**
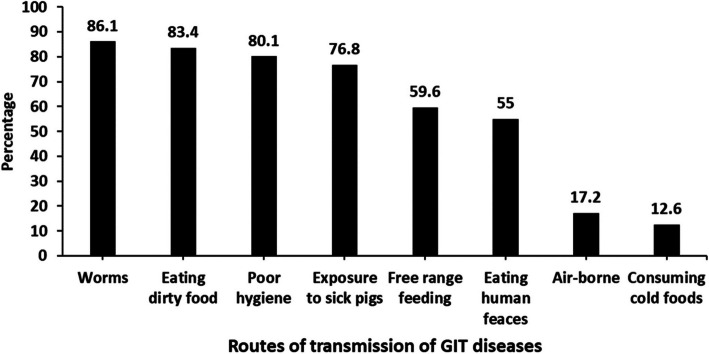
Routes of transmission of GIT diseases as perceived by the farmers from Masindi Biosecurity measures practiced by farmers to prevent GIT disorders of pigs

Similarly, the correct transmission routes for GIT disease as identified by farmers were eating dirty foods (83.4%), poor hygiene (80.1%), exposure to sick pigs (76.8%), free range rearing (59.6%) and eating human feces (55%). Majority (86.1%) of farmers associated worms with transmission of diseases in their pigs (Fig. 3).

The majority of farmers confined the pigs to prevent GIT disease (70.6%; CI: 63.1–77.3), treated the sick pigs (62.9%; CI: 55.2–70.2), and sold the sick animals (40.6%; CI: 33.1–48.4) and only (25.9%; CI: 19.5- 33.2) consult a veterinarian as in Table 3.

**Table 3:** Biosecurity practices to prevent GIT diseases of pigs by farmers

Practices	Frequency (*N* = 170)	Percentage %	Confidence Interval (CI) 95%
Confinement of the pigs	124	70.6%	63.1–77.3
Buy drugs and treat the sick pig by self	107	62.9%	55.2- 70.2
Sell the sick animal to buyers	69	40.6%	33.1- 48.4
Use herbs to treat the sick pig	52	30.6%	23.8- 38.1
Call the veterinarian for advice	44	25.9%	19.5- 33.2
Hide the pigs away	41	24.1%	17.9- 31.3
Stop buying other pigs	38	22.4%	16.3- 29.4
Slaughter the sick pig	6	3.5%	1.3- 7.5

### Bivariate analysis of factors associated with pig gastrointestinal disorders in Masindi district

#### Socio-demographic factors associated with GIT disorders in pigs

The bivariate analysis to determine risk factors associated with GIT disorders at the farm was carried out on the following factors; i) demographic factors: gender, religion, education level, marital status, place of residence, activities in the value chain and duration of rearing pigs; ii) general husbandry practices are: breeds, management, production, housing, mixing of pigs with other herds and sharing of breeding boars, and iii) feeds and feeding practices include feed categories (raw, cooked, dried feeds or mixed with additives), scavenging for food, supplementing with household food left overs, feeding with swill or with factory feeds.

Gender was the only socio-demographic factor significantly associated with GIT disorders. It was observed that female farmers were more than 2 times likely to be associated with occurrence of GIT disorders (cOR 2.257; *p* = 0.04) than their counterpart-male farmers. Also, farm owners were more than 2.5 times likely to report GIT disorders than the farm laborers (cOR2.897, *p* = 0.06), though this was not significant (Table 4).

**Table 4:** Socio-demographic characteristics of farmers as risk factors for GIT disorders in pigs

	Experienced cases of GIT diseases on the farm				
	Frequency/percentage				
**Demographics**	Attribute	Yes n(%)	No n(%)	cOR	*p*- value
Sex of the respondent	Male	62(36.5%)	23(13.5%)	ref	
	Female	73(42.9%)	12(7.1%)	2.257	0.04*
Religion of the respondent	Catholic	57(33.5%)	11(6.5%)	ref	
	Protestant	54(31.8%)	20(11.8%)	0.521	0.121
	Others	24(14.1%)	4(2.4%)	1.158	0.817
Education level of respondent	None	10(5.9%)	2(1.2%)	ref	
	A level	9(5.3%)	2(1.2%)	0.9	0.924
	Degree holder	8(4.7%)	1(0.6%)	1.6	0.72
	Primary	60(35.3%)	21(12.4%)	0.571	0.492
	Dip holder	8(4.7%)	1(0.6%)	1.6	0.72
	O level	40(23.5%)	9(5.3%)	1	1
Marital status	Single	25(14.7%)	10(5.9%)	ref	
	Widowed	12(7.1%)	1(0.6%)	4.8	0.156
	Married	92(54.1%)	23(13.5%)	1.673	0.246
	Divorced	6(3.5%)	2(1.2%)	1.2	0.839
Place of residence	Rural	118(69.4%)	33(19.4%)	ref	
	Urban	17(10%)	2(1.2%)	2.377	0.263
Activities in the value chain	Laborer	9(5.3%)	6(3.5%)	ref	
	Owner	126(74.1%)	29(17.1%)	2.897	0.06
Duration of rearing pigs	< 5 years	87(51.2%)	25(14.7%)	ref	
	≥5 years	48(28.2%)	10(5.9%)	1.379	0.349

*cOR* crude odds ratio

Significant *p*<0.05

#### Pig husbandry factors associated with GIT disorders

The following general farm practices were significantly associated with GIT disorders; farmers who kept local breeds of pigs were three times more likely to experience GIT disorders (cOR 3.078; *p* = 0.005), than keeping exotic breeds (Table 5). Also, rearing grower pigs (porker to finisher) were equally at a higher risk (cOR 3.078; *p* = 0.024) than farmers keeping breeder pigs (piglets to weaner) or those with mixed age groups. A higher percentage of GIT cases were reported among pigs which mix with other herds 70(41.2%); these were 3 times more likely to experience GIT disorders (cOR 3.635, *p* = 0.003) than the confined pigs 65(38.2%). On the other hand, rearing pigs in temporary (cOR 0.253; *p* = 0.007) or permanent houses (cOR 0.229; *p* = 0.008), was more protective against GIT disorders than pigs without any shelter or the rope (Table 5).

**Table 5:** Pig husbandry factors associated with occurrence of Gastrointestinal disorders

	Ever experienced cases of GIT diseases on the farm				
	Frequency/percentage				
	Attribute	Yes	No	cOR	*p*- value
**General farm practices**					
Breed of pigs kept	Local	106(62.4)	19(11.2)	3.078	0.005^*^
	Exotic/crosses	29(17.1)	16(9.4)	ref	
Ownership of the pigs	Family business (group owned)	47(27.6)	15(8.8)	0.712	0.38
	Individually owned	88(51.8)	20(11.8)	ref	
Management system	Intensive/confined	51(30)	27(15.9)	0.315	0.149
	Tethering	72(42.4)	6(3.5)	2	0.428
	Communal/free range	12(7.1)	2(1.2)	ref	
Production system	Piglet to weaner (breeder only)	2(1.2)	2(1.2)	0.326	0.273
	Porker to finisher (Grower only)	44(25.9)	4(2.4)	3.584	0.024*
	Mixed	89(52.4)	29(17.1)	ref	
Do your pigs mix with other herds	Yes	70(41.2)	8(4.7)	3.635	0.003*
	No	65(38.2	27(15.9)	Ref	
Do you share a boar with other farmers	Yes	95(55.9)	25(14.7)	0.95	0.903
	No	40(23.5)	10(5.9)	ref	
Type of housing system	Temporary	47(27.6)	18(10.6)	0.253	0.007^*^
	Permanent	26(15.3)	11(6.5)	0.229	0.008^*^
	None	62(36.5)	6(3.5)	ref	
**Feeds and feeding practices**					
Raw tubers	Yes	101(59.4%)	19(11.2%)	2.502	0.018^*^
	No	34(20%)	16(9.4%)	ref	
Cooked feeds	Yes	84(49.4%)	14(8.2%)	2.471	0.018^*^
	No	51(30%)	21(12.4%)	ref	
Drying of feeds	Yes	10(5.9%)	2(1.2%)	1.28	0.727
	No	125(73.5%)	33(19.4%)	ref	
Pigs scavenging for food	Yes	50(29.4%)	2(1.2%)	9.706	< 0.001^*^
	No	85(50%)	33(19.4%)	ref	
Supplement with household food left overs	Yes	89(52.4%)	20(11.8%)	1.451	0.334
	No	46(27.1%)	15(8.8%)	ref	
Swill feeds from hotel	Yes	6(3.5%)	2(1.2%)	0.767	0.752
	No	129(75.9%)	33(19.4%)	ref	
Feeds from the factory	Yes	73(42.9%)	21(12.4%)	0.785	0.53
	No	62(36.5%)	14(8.2%)	ref	
Others specified feeds: (grass and weeds)	Yes	55(32.4%)	20(11.8%)	0.516	0.082
	No	80(47.1%)	15(8.8%)	ref	

*cOR* crude odds

^*^Significant *p* < 0.05

In respect to feeds and feeding practices, the pigs that were fed on raw tubers and cooked foods were 2.5 (cOR 2.502; *p* = 0.018) and 2.47 times (cOR 2.471; *p* = 0.018), more likely to be associated with GIT disorders, respectively. Also, scavenging pigs were significantly associated with occurrence of GIT (cOR 9.706; *p* < 0.001); as such pigs were nine times more associated with GIT disorders than other categories (Table 5).

### Multivariable analysis to determine independent factors associated with GIT disorders in pigs

The bivariate analysis generated a number of significant risk factors: gender, mixing of herds pigs, production system, feeds preparation, feeding practices and type of housing system which were selected for the multivariable analysis using a binary regression model (Table 6).

**Table 6:** Multivariable logistic regression analysis of the variables associated with GIT disorders in pigs

	Experienced cases of GIT diseases on the farm				
	Frequency/percentage				
**Variable**	Attribute	Yes n(%)	No n(%)	aOR	*P* value
Sex of the respondent	Male	62(36.5%)	23(13.5%)	Ref	
	Female	73(42.9%)	12(7.1%)	3.163	0.018^*^
Production system	Breeder only	2(1.2)	2(1.2)	0.111	0.073
	Grower only	44(25.9)	4(2.4)	1.222	0.774
	Breeder and growers	89(52.4)	29(17.1)		
Do your pigs mix with other herds	Yes	70(41.2)	8(4.7)	4.141	0.015^*^
	No	65(38.2	27(15.9)		
Type of housing system	Temporary	47(27.6)	18(10.6)	1.919	0.502
	Permanent	26(15.3)	11(6.5)	2.292	0.465
	None	62(36.5)	6(3.5)		
Raw root tubers	Yes	101(59.4%)	19(11.2%)	2.703	0.017^*^
	No	34(20%)	16(9.4%)		
Cooked feeds	Yes	84(49.4%)	14(8.2%)	2.081	0.074
	No	51(30%)	21(12.4%)		
Pigs scavenging for food	Yes	50(29.4%)	2(1.2%)	9.605	0.003^ss^
	No	85(50%)	33(19.4%)		

*aOR* adjusted odds ratio

^*^Significant association, *p* < 0.005

Of these, the gender of respondent was significantly associated with occurrence of GIT disorders (*p* = 0.018, OR = 3.163, CI: 1.213, 8.244). Female farmers were 3.163 times more likely to have experienced cases of GIT diseases on the farm as compared to their male counterparts. The mixing of herds between farms was a significant factor for GIT disorders (*p* = 0.003, aOR = 4.141, 95%CI (1.317,13.013). Such farms were 4.141 times more likely to experience cases of GIT disorders compared to those whose pigs were confined.

Similarly, pigs fed on raw tubers were significantly associated with GIT disorders (*p* = 0.017, aOR = 2.703, 95% CI (1.198,6.099). Pigs fed on raw root tubers were almost 3 times more likely to experience GIT disorders than those on other feeds. Also, scavenging pigs were significantly associated with occurrence of GIT (*p* < 0.001, aOR = 9.605, 95%CI (2.131,43.289). Pigs scavenging for food were almost 10 times more likely to experience GIT cases than non-scavenging pigs (Table 6).

## Discussion

Our focus was to investigate the GIT disorders of pigs in general, among smallholder farmers in Masindi district of Uganda, as a supplement to previous researches that addressed specific pathogens and parasites associated with GIT [3–5, 7, 29–32]. Moreover, these studies excluded Masindi district, and yet, the district lies within the greater oil region of Uganda, where piggery production is targeted as priority investment sector for the smallholder farmers.

That a larger proportion (72%) of the smallholder farmers reported the occurrence GIT disorders, strongly affirms the looming health challenge affecting the health and productivity of pig in the district and Uganda at large. This is consistent with the recent study in the three districts of Masaka, Mityana and Mpigi which reported the proportion of 50–51.7% [3]. The presence of GIT disorders is main predictor of the losses incurred by the pig enterprises; because of the compromised physiological functions of the GIT. Evidently, GIT disorders interfere directly with digestion, nutrient absorption, feed conversion efficiency and ultimate growth rates in the affected pigs [9, 10, 13]. Obviously, the smallholder farmers will incur additional losses due to extra feeding in attempt to hasten the growth rate, increasing the overall operational costs. In essence, husbandry practices that sustain a stable microflora ecosystem and optimal GIT homeostasis are desirable in curbing GIT disorders for improved pig health and productivity.

It was also reported that GIT disorders affected the weaners and piglets more than adult pigs. The observed occurrence of diseases in weaners and suckling piglets may be linked to the age-related immunity that is acquired as piglets grow into adult pigs [33–35]. This is consistent with previous reports among young pigs in various parts of Uganda [29, 30] and elsewhere [31]. On the other hand, it is also probable that the older pigs were equally affected but did not present clinical signs due to age-related immunity, giving the impression that they were least affected.

Farmers observed GIT disorders throughout the year, with slightly higher cases in the dry season although this was not significant. The relatively higher incidences of GIT disorders in the dry season can be attributed to the changes in agricultural activities and management systems that promote scavenging since there is plenty of crop residues left in the gardens after the harvest [32, 36]. On the contrary, the farmers restrict pig movement to protect the planted crops from damage by the scavenging pig, during the rainy season [32]. Yet, other farmers continue to release their pigs to graze on abundant green herbage around the homesteads during the rainy season, which may explain the sustained high occurrence of GIT disorders throughout the year. This observation is consistent with the study from Ethiopia which reported the grazing of pigs occurs both during the dry and wet seasons of the year [36]. Such a practice certainly increases the risk of disease transmission and spread, as pigs come in contact with potentially contaminated and infectious food resources in the environs.

The study revealed that a large proportion of the farmers were knowledgeable of major clinical signs associated with GIT disorders. Knowing clinical signs and symptoms is crucial for detection of the disease at an early stage in order seek treatment before the situation worsens. This was consistent with the other studies [37, 38] that reported the importance of farmers knowledge in early detection of the diseases on the farm. Conversely, lack of adequate knowledge of diseases is one of the main contributors to the vicious cycle of endemic diseases in animals and humans prevalent in poor rural communities in Africa [39, 40].

The farmers exhibited good knowledge of the potential routes of transmission of GIT diseases, which is crucial in avoiding and preventing the spread of GIT disorders on their farms. Evidently, having the basic knowledge of the routes of transmission for any given disease, enables farmers to apply preventive measures towards the prevailing disease [41]. However, this should not be construed to mean that farmers would always comply with these measures to minimize the prevent or the control the spread of GIT disorders on the farm. On the whole, smallholder farmers are encouraged to adopt biosecurity measures, if they are to reduce the introduction and spread of pathogens on their farms. Plausibly, implementation of farm biosecurity measures enables the farmers to prevent and control known and unknown health challenges [41–43] as a means to sustainable pig production.

A significant proportion of the farmers sold the sick pigs in attempt to lessen economic losses. Of concern, such only increases the risk of disease spread in the community, as the practice breaches the core biosecurity principles. Whereas biosecurity issues may appear important, they are not in conformity with the farmers’ priorities of safeguarding family livelihoods and earning household income. Undeniably, most smallholder farmers find it challenging to adopt biosecurity measures because of the high cost, socio-cultural biases and poor veterinary services [44]. Thus, implementation of biosecurity guidelines will require inclusive policies and educative programs that secure the social economic wellbeing of the farmer as apriority, and conscious of their unique social and cultural context [45].

It can be noted that half of the respondents in this study were female, engaged as owners of the pig farm. This was similar to an earlier study in Mukono, Masaka and Mpigi, where more than half of the farmers were also women [3]; signifying the main role women played in pig production in Uganda. Arguably, in the male-dominated communities of Uganda, cultural norms dictate the rearing of large livestock such as cattle as a male domain [46, 47]. Meanwhile, lower social prestige animals such as pigs and chicken are relegated to the care, ownership and sale by women [47–49].

It can be surmised that the association of female farmers with GIT disorders, rather than being a risk factor, signifies their close interaction with the pigs and deeply involved in the day-to-day husbandry practices. A plausible explanation is that female farmers tend to be keener at observing signs of ill-health and report the incidences more frequently, than their counterpart male farmers, who often overlook such details. This finding is in consistent with the health-reporting behaviors of women, who tend to observe and report minor health problems as compared to men [50]. Thus, female famers should be considered equally when designing GIT disorder control strategies and others for pig husbandry practices as their male counterparts.

We observed that pig fed on raw tubers especially cassava and/or sweet potatoes were significantly associated with GIT disorders. This is a controversial finding since pigs are known to eat raw cassava and sweet potatoes without any known complications. Nonetheless, future investigation is required to confirm or rule out this observation. The reported increase in GIT disorders among pigs which comingle with other herds or among scavenging pigs was due to increased chances of contracting infections from other sick pigs or in the environment. This is a plausible explanation for the 4.1 and almost 10 times likelihood for the occurrence of GIT disorders among the herds of pigs that were comingling or scavenging for food, respectively. Also, this concurs with previous findings of free ranging system and scavenging in pigs increased the risk of exposure to various diseases that affect production and of zoonotic nature [7, 36, 44, 51–54].

Although housing was major protective factor against GIT disorders in pigs at bivariate analysis, this association was not significant at multivariate analysis. This finding is contrary to the previous observation of the risk associated with comingling and scavenging in pigs which increase the risk of GIT health related problem in Kenya [55]. From practical observations, pigs which are kept on a rope or freely roaming, would be exposed to higher risks of GIT disorders and hence confinement and housing for pigs is recommended for smallholder farmers. Notwithstanding, free-range system remains popular and is widely practiced in a number of African countries including Kenya [52, 55], Rwanda [56], Ethiopia [36] and Zambia [57], among others. Farmers opt for free ranging system because they incur less cost and on labor of feeding and housing such pigs [32]. It must be stressed that GIT disorders in pigs are frequently the result of multiple causes and rarely due to the effects of a single factor. This study however, did not investigate the specific causes of GIT disorders but instead relied on the clinical history and symptoms as reported by the farmers.

## Conclusion

This study shows widespread occurrence of GIT disorders in pigs raised by smallholder farmers in Masindi which inevitably affects productivity and profitability of the pig enterprise. Involving women as they are key partners in the pig health control programs is suggested. Scavenging pigs, and those that comingle with other pigs were at a higher risk of GIT disorders, a practice which should be discouraged. Farmers should be encouraged to adopt confinement and housing of pigs as good husbandry practices. These results are relevant in guiding smallholder pig farmers towards better husbandry practices as a means to achieve improved pig health and production. Future programs designed to prevent GIT disorders should be cognizant of the unique social and cultural context of the community involved.

## Supplementary Information

Supplementary Material 1.

## Data Availability

The datasets analysed during the current study are available from the corresponding author on reasonable request.

## Abbreviations

A’ Level: Advanced level of Education (higher secondary)
aOR: Adjusted odds ratio
CI: Confidence Interval
cOR: Crude odds ratio
GIT: Gastrointestinal
O’ Level: Ordinary level of Education (lower secondary)
