# Supplementary material for: Assessing the knowledge and practices of smallholder pig farmers and associated risk factors for swine gastrointestinal disorders in Masindi district, Uganda
Source: BMC Vet Res. 2025 Mar 31;21:228. doi: 10.1186/s12917-025-04667-2 (PMC11956471; doi:10.1186/s12917-025-04667-2)
Supplement: Supplementary file 1 — Supplementary Material 1. [file 12917_2025_4667_MOESM1_ESM.pdf]

Date……………………   ID No.

QUESTIONNAIRE

Harnessing microbial probiotics for improving pig health and productivity

Introduction

A major challenge to raising pigs are high cost of the feeds that constitute more than two-thirds of the total operational costs, thus a need for feed conversion efficiency to enable rapid growth. This can be achieved if the gastrointestinal symbiotic microbial ecosystem is balanced. The research study is to improve pig health and productivity using probiotics in Uganda specifically to assess the prevailing community's knowledge and practice in managing various gastrointestinal disease of economic importance to the pig industry.

1.0 General iformation

Do you agree to participate in this study? Yes……………………No……………………

District………………………County……………………..Sub-county………………………..

Parish…………………………..Village……………………

1.0 Household characteristics

1.1	Sex	1. Male<br>2. Female
1.2	Religion	1. Catholic<br>2. Protestant<br>3. Traditional<br>4. Others(Specify

**1.3**	Education level of Respondent	1. None<br>2. Primary<br>3. O' level<br>4. A' level<br>5. Dip. Holder<br>6. Degree holder<br>7. Other Specify
**1.4**	Marital status	1. Single<br>2. Married<br>3. Widowed<br>4. Divorced
**1.5**	Residence	1. Rural<br>2. Urban
**1.6**	Activities in the pig value chain	1. Farmer/owner<br>2. Laborer<br>3. Butcher

		4. Slaughter house worker<br>5. Slaughter house owner<br>6. Others
**1.7**	Keep other animals at home	1. Yes<br>2. No

2.0 Farm characteristics

2.1	Duration of rearing the pigs	…………………………………Year(s)	
2.2	Other animal types and numbers	Type	Number
		1. Cattle<br>2. Goat<br>3. Sheep<br>4. Pig<br>5. Dogs	…………..<br>…………..<br>…………..<br>…………..<br>…………..

**2.3**	Management system	1. Intensive/confined<br>2. Tethering<br>3. Communal/free range
**2.4**	Production system	1. Only breeder<br>2. Only grower<br>3. Breeder and grower
**2.5**	Housing system	1. Temporary<br>2. Permanent<br>3. Semi-permanent<br>4. No available
**2.6**	Do your pigs mix with other herds	1. Yes<br>2. No
**2.7**	Share boars with other farmers	1. Yes<br>2. No
	Type of labor	1. Family labor<br>2. Hired labor

3.0 Knowledge, attitudes and Practices relating to managing GIT diseases in pigs

**3.1**	**Knowledge**	
3.2	Do you know any of GIT diseases in pigs?	1. Yes<br>**2.** No
3.3	What is the local names of GIT diseases that you know?	………………………………………………..
3.4	Have you experienced cases of GIT diseases on	1. Yes

	your farm?	2. No		
3.5	If yes, how do animals get GIT diseases?	………………………………………………<br>………………………………………………<br>………………………………………………<br>……………………………………………….<br>……………………………………………….		
3.6	Which age group of the pigs commonly suffer from GIT diseases?	1. Piglets<br>2. Weaners<br>3. Adults		
3.7	Breed of pigs commonly affected by GIT diseases	1. Local<br>2. Improved<br>3. Both		
3.8	What signs can you use to tell that the animal has GIT diseases?	……………………………………………….<br>……………………………………………….<br>……………………………………………….<br>…………………………………………………		
4.0	**What are some of the ways through which GIT diseases can be transmitted in pigs?**	Yes	No	I don't Know
	i. Exposure to sick pigs			
	ii. Eating dirty or contaminated food			
	iii. Poor hygiene			
	iv. Worms			
	v. Consuming of cold food/water			
	vi. Air borne			
	vii. Free range feeding			
	viii. Human feces			
	ix. Others (specify)			
4.1	Common season for the occurences of GIT diseases in pigs			
	1. Dry season			
	2. Wet season			
	3. Both dry and wet seasons			
4.2	**Signs of GIT diseases in pig**	**Yes**	**No**	**I don't Know**
	i. Fever			
	ii. Intense weakness			
	iii. Diarrhea			
	iv. Poor hair coat			
	v. Poor appetite			
	vi. Death			
	vii. Weigh loss			
	viii. Vomiting			
	ix. Salivation			
	Others (specify)			

4.3	**What are some of the ways you can use to prevent GIT diseases in pigs**	Yes	No	List what is used
	i. Call Veterinary Doctor			
	ii. Treat pigs myself using drugs available			
	iii. Herbs/local remedies			
	iv. No treatment			
	v. Vaccination			
	vi. Others (Specify)			
4.4	Do you think GIT dieases in pigs is a public health risks	Yes	No	Don't know
4.5	Know any of the obstacles to prevent pig owners from using control methods			
5.0	**Practices**			
5.1	Have you sold a pig or pig products with GIT disease signs			
5.2	Control methods used when pigs suffer from GIT			
	1. Confine pigs<br>2. Hide pigs away from house or other pigs<br>3. Give drugs<br>4. Stop buying<br>5. Tether<br>6. Call a vet<br>7. Use herbs			

Thank you
